# Experimental and Numerical Study on Stimulated Brillouin Scattering in a Spun Optical Fiber

**DOI:** 10.3390/s25041127

**Published:** 2025-02-13

**Authors:** Ester Catalano, Agnese Coscetta, Raffaele Vallifuoco, Luigi Zeni, Aldo Minardo

**Affiliations:** Department of Engineering, Università della Campania Luigi Vanvitelli, Via Roma 29, 81031 Aversa, Italy; ester.catalano@unicampania.it (E.C.); agnese.coscetta@unicampania.it (A.C.); raffaele.vallifuoco@unicampania.it (R.V.); luigi.zeni@unicampania.it (L.Z.)

**Keywords:** stimulated Brillouin scattering, spun optical fibers, temperature sensors, strain sensors

## Abstract

This paper presents a numerical and experimental investigation of stimulated Brillouin scattering (SBS) in a highly birefringent spun optical fiber. When subjected to bending, the variation in the state-of-polarization (SOP) of the pump and probe waves induces a periodic oscillation of the Brillouin gain, with a period equal to the elliptical birefringence of the fiber. The experiments were corroborated by numerical simulations, combining the coupled equations governing the SOP evolution in bent spun fibers, with a scalar SBS model valid for Brillouin optical frequency-domain analysis (BOFDA) sensors.

## 1. Introduction

Stimulated Brillouin scattering (SBS) is a nonlinear optical effect easily observed in single-mode optical fibers, owing to the large optical intensity in their core. Since its first observation in optical fibers [1], it was recognized early that SBS can be exploited as a sensing mechanism for temperature and strain measurements, based on the sensitivity of the so-called Brillouin Frequency Shift (BFS) from these measurands [2]. The BFS corresponds to the frequency shift between a pump and a counter-propagating probe wave, which results in the maximum power coupling between the two waves. Conventional SBS-based sensors cannot distinguish the variation in strain and temperature, as both affect the BFS in a similar manner. Several methods have been proposed to solve this issue, including those relying on specialty fibers such as photonic crystal fibers [3], suspended-core fibers [4], and dispersion-shifted fibers [5]. One of the most efficient methods to discriminate strain and temperature relies on the measurement of two uncorrelated parameters of the fiber, namely, the BFS and local birefringence in a polarization-maintaining (PM) fiber. Such an approach has been successfully demonstrated in a PANDA fiber [6], resulting in a complete and accurate separation of temperature and strain effects. However, this approach has the drawback of requiring the excitation of the so-called Brillouin dynamic gratings (BDGs) to retrieve the local birefringence. In brief, a BDG is excited by injecting two frequency-shifted pump waves at the two ends of the fiber, both aligned along one of the two eigenmodes of the PM fiber. These two pump waves generate, through electrostriction, an intense acoustic wave (the BDG), provided that their frequency shift corresponds to the BFS of the fiber. An orthogonally polarized probe wave is then injected into one end of the same fiber. If the probe wave is frequency-shifted with respect to the pump by a precise quantity related to the fiber birefringence, it will be efficiently backscattered by the BDG, resulting in a measurable backscattered light. Thus, the birefringence can be retrieved by monitoring the intensity of the backscattered light while varying the frequency shift between the pump and probe waves. This procedure is quite complex and requires a precise polarization control of the injected beams. Furthermore, the frequency shift between the pump and probe waves lies in the range of several tens of GHz [6]; thus, two separate lasers are usually necessary to generate the required optical frequencies. The above considerations suggest that an alternative method to measure the birefringence in PM fibers would be desirable, by which temperature and strain could be separated without adding complexity to the measurement system.

In this work, we demonstrate, both numerically and experimentally, that the elliptical birefringence of a bent spun fiber can be retrieved using conventional Brillouin optical frequency-domain analysis (BOFDA) measurements. In detail, we show that the elliptical birefringence induces a periodical undulation of the Brillouin gain, whose period corresponds to the beat length of the elliptical birefringence, while its amplitude grows with the curvature of the fiber. As both birefringence and BFS vary with temperature and strain, measuring both parameters provides a mechanism to separate these measurands.

In the following sections, we report the results of a numerical study, anticipating the appearance of Brillouin gain undulations in bent spun fibers. Then, we experimentally confirm the presence of these undulations and characterize their dependence on temperature and strain. The conclusions follow.

## 2. Numerical Modeling of the SBS Interaction Along a Spun Fiber

**Figure 1:**
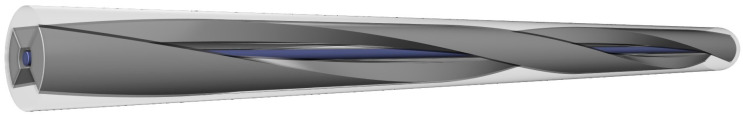
Schematic representation of one spin period of the bow-tie spun fiber used for experiments.

In the following, we consider a highly birefringent spun fiber fabricated by drawing the fiber from a spinning highly birefringent (bow-tie) preform, thus forming a built-in rotation of the principal axes (see Figure 1).

**Figure 2:**
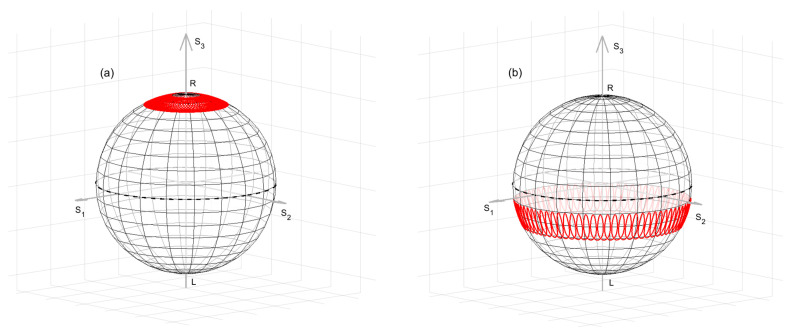
Representation on a Poincaré sphere linked to the laboratory coordinate system of the SOP trajectory in a straight spun fiber, with $L_{S}=\;4mm$ and $L_{b}=12.2\;mm$. The input SOP corresponds to one of the two fiber eigenmodes (**a**), or to the sum of the two fiber eigenmodes (**b**). *L* and *R* represent the left- and right-handed circular polarizations, respectively.

The spun birefringent optical fiber is characterized by two parameters: the spin period $L_{s}$ and the linear birefringence beat length of the unspun birefringent precursor fiber $L_{b}$ [7]. As the principal axes change their direction along the spun fiber, there are no polarization eigenstates in the laboratory coordinate system. However, in the helical coordinate system linked with the fiber principal axes, there exist two orthogonal helical polarization modes, whose state-of-polarization (SOP) remains unchanged. On the Poincaré sphere linked to the helical coordinate system, these two elliptical eigenmodes are found by drawing the birefringence vector $\vec{\Omega }$ given by [8]

(1) $$\vec{\Omega }=\vec{\Delta \beta }+\vec{\alpha }$$

where $\vec{\Delta \beta }$ is the linear birefringence vector with magnitude $∆\beta =2\pi /L_{b}$ and direction $\vec{s_{1}}$, while $\vec{\alpha }$ is the circular birefringent vector with magnitude $2\xi =4\pi /L_{S}$ and direction $\vec{s_{3}}$ (see Figure 2). In the laboratory coordinate system, the local linear birefringence vector $\vec{\Delta \beta }$(z) rotates in the equator plane with an angular rate 2ξ.

Spun fibers are also characterized in terms of a dimensionless parameter $\sigma =L_{S}/L_{b}$. In this work, we focus our attention on spun fibers with a small σ ($L_{s}\ll L_{b}$). As the ellipticity of the two fiber eigenmodes is $\sqrt{\sigma^{2}+1}-\sigma$ [8], spun fibers with a small σ have two quasi-circular fiber eigenmodes.

In absence of Faraday effect, the SOP evolution of a monochromatic field propagating along a spun fiber is governed by the following equations [9]:

(2) $$\begin{array}{c} \frac{dE_{L}}{dz}=i\left[\left(\frac{∆\beta }{2}\right)\exp\left(i2\xi z\right)+\left(\frac{\delta }{2}\right)\right]E_{R},\\ \frac{dE_{R}}{dz}=i\left[\left(\frac{∆\beta }{2}\right)\exp\left(i2\xi z\right)+\left(\frac{\delta }{2}\right)\right]E_{L}. \end{array}$$

where $E_{L}$ and $E_{R}$ are the electric fields with the left- and right-hand circular polarizations, respectively, and δ is the phase shift induced by bending. In absence of bending (i.e., δ=0), the propagation constants of the two eigenmodes differ by ${\Delta \beta }_{c}=2\pi /L_{c},$ where $L_{c}$ is the elliptical polarization beat length given by [9]

(3) $$L_{C}=\frac{L_{b}L_{S}}{\sqrt{4{L_{b}}^{2}+{L_{S}}^{2}}-2L_{b}}$$

To visualize this process, we represent in Figure 2a the SOP evolution across the Poincaré sphere linked to the laboratory coordinate system, as obtained by solving Equation (2) using the function ode45 of MATLAB R2024b. For our calculations, we considered a straight spun fiber with $L_{S}=4\;mm$ and $L_{b}=12.2\;mm$ (which results in an elliptical beat length $L_{c}=15\;cm$), and an input SOP corresponding to one of the two polarization eigenmodes of the fiber. The figure reveals that the SOP evolves along cycloids around the polarization eigenmode, with a revolution period equal to $L_{c}$.

Let us now consider an input SOP equal to the sum of the two eigenmodes of the spun fiber. On the Poincaré sphere linked to the helical coordinate system, such SOP corresponds to the vector perpendicular to $\vec{\Omega }.\;$The newly calculated SOP evolution is shown in Figure 2b. In this case, the SOP stays almost linear (the SOP trajectory moves close to the Poincaré sphere equator), while slowly rotating along the fiber with a period equal to $L_{C}$.

As a next step, we consider the SOP evolution of two counterpropagating beams (the “pump” and “probe” fields in the SBS interaction), with the pump propagating along the +z direction, and the probe propagating along the −z direction. In the following, we neglect the birefringence difference between the two waves, as their frequency difference is relatively small (≈10 GHz). Furthermore, the fiber is supposed to be relatively short (L<<1 km). Therefore, we can safely neglect the SBS-induced power coupling influence on the SOP evolution of the two beams (the so-called “polarization pulling” effect), which, in conventional silica fibers, becomes significant for lengths of several kilometers [10,11].

**Figure 3:**
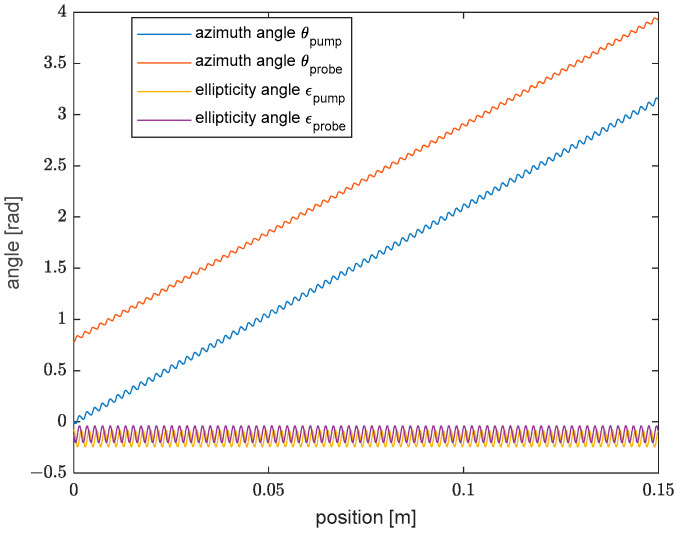
Azimuth θ and ellipticity ϵ angle of the pump and probe polarizations, computed for a fiber length equal to $L_{C}$. The input SOP of the pump corresponds to the sum of the two fiber eigenmodes, while the input SOP of the probe has an azimuth angle rotated by π/4.

Under these hypotheses, the SOP evolution of the pump and probe waves are equally described using Equation (2), with the only difference of a minus sign at the right-hand side of both equations when calculating the SOP evolution of the probe wave [8]. Let us suppose now that the input SOP of the pump corresponds to the sum of the two fiber eigenmodes (as in Figure 2b), while the input SOP of the probe has the same ellipticity angle (ϵ = −0.08 rad), but an azimuth angle shifted by π/4. As we will see later, this choice is optimal when one wants to measure the elliptical birefringence of the fiber through SBS measurements. Based on these assumptions, the SOP of the two beams will follow the same trajectory represented in Figure 2b, but with a different starting point. For the sake of clarity, we show in Figure 3 the evolution along a fiber length equal to $L_{C}$ of the azimuth θ and ellipticity ϵ angles of the pump and probe polarizations, calculated using [12]

(4) $$\begin{array}{c} \theta (z)=-\frac{1}{2}arg\left(E_{r}(z)/E_{l}(z)\right)\\ \epsilon (z)={tan}^{-1}\frac{\left|E_{r}(z)/E_{l}(z)\right|-1}{\left|E_{r}(z)/E_{l}(z)\right|+1} \end{array}$$

From Figure 3, we observe that the azimuth angle of the pump and probe varies by 2π, while their ellipticity angle remains small and constant on average. Also, both angles exhibit a small sinusoidal oscillation with a period equal to half the spin period (2 mm, in our case). Other simulations, not shown here, revealed that, by changing the input SOP, the azimuth and ellipticity angles follow the same behavior represented in Figure 3, with the only difference of a larger amplitude of the azimuth angle oscillations at increasing ellipticity.

As a next step, we consider the case of a bent spun fiber (i.e., δ≠0). The phase delay induced by bending was calculated using $\delta =2\pi /L_{ind}$ with $L_{ind}=22.792\frac{\lambda_{0}}{n^{3}}\frac{R^{2}}{r^{2}}$ [7], where n is the average refractive index of the fiber, $\lambda_{0}$ is the optical wavelength, R is the radius of curvature, and r is the external radius of the fiber. In the following, we consider a radius of curvature R ranging from 100 cm down to 3 cm, while r = 62.5 µm.

**Figure 4:**
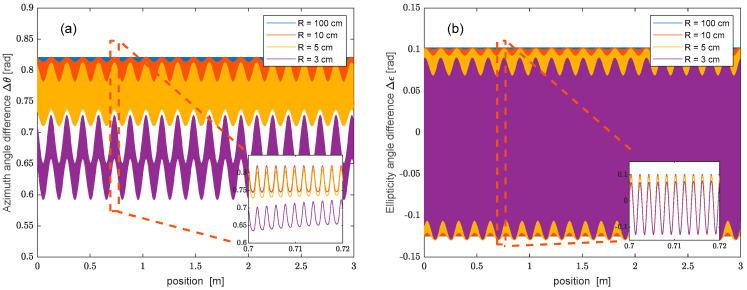
Difference between the azimuth (**a**) and ellipticity (**b**) angles of the pump and probe polarizations, computed for a 3 m long spun fiber. The input SOP of the pump corresponds to the sum of the two fiber eigenmodes, while the input SOP of the probe has an azimuth angle rotated by π/4.

To illustrate the effect of curvature, it is convenient to calculate the difference between the azimuth and ellipticity angles of the pump and probe polarizations, at varying curvature radii. The results, reported in Figure 4 for a fiber length of 3 m, reveal that, at increasing curvatures, the difference between the azimuth and ellipticity angles acquires an increasing undulation with a period equal to $L_{C}$ (15 cm in our simulations). These results are consistent with previous studies of the SOP evolution in bent spun fibers (e.g., Refs. [7,13]). For example, Ref. [13] presents evidence that spun fiber bending induces a coupling between its two eigenmodes, which manifests itself as a long-period undulation in the average SOP with an amplitude increasing with the curvature. Figure 4a,b highlight the presence of fast oscillations with a period of 2 mm, i.e., half the spin period.

In the following, we employ the computed SOP trajectories of the pump and probe fields to calculate the SBS gain variations along the spun fiber. As mentioned before, for relatively short fibers (*L* << 1 km), the SBS polarization pulling effect can be safely neglected. This permitted us to employ a *scalar* SBS model, where the influence of the pump and probe polarization states is accounted for by simply adopting an SBS mixing efficiency $\eta_{SBS}$ [14]:

(5) $$\eta_{SBS}\left(z\right)=\frac{1}{2}\left(1+{\vec{s}}_{pump}\left(z\right)\cdot {\vec{s}}_{probe}\left(z\right)\right)$$

where ${\vec{s}}_{pump}$ and ${\vec{s}}_{probe}$ are the Stokes parameters (i.e., the SOP coordinates on the Poincaré sphere) of the pump and probe waves, when seen from the same direction. Let us recall that, in a BOFDA sensor, a pump wave with a small ac intensity modulation interacts with a counterpropagating probe wave, provided that their frequency shift $\Delta_{\nu pp}$ is close to the local Brillouin frequency shift $\nu_{B}$ of the fiber [15]. Under these circumstances, an intensity modulation is impressed on the probe wave, which is conveniently detected at the output of the fiber through a vector network analyzer (VNA). The ratio between the ac intensities of the probe and pump waves constitutes the so-called baseband transfer function $TF\left(\omega_{m}\right)$ of the fiber. By acquiring $TF\left(\omega_{m}\right)$ over a proper range of frequencies $\omega_{m}$, the spatial profile of the Brillouin gain distribution can be recovered using inverse Fourier transform [15].

Under the undepleted-pump approximation, the baseband transfer function can be expressed as [16]

(6) TFωm=ESOL∫0LESOzgB,acz,ωme−2jωmncdz

where $E_{SO}\left(z\right)$ is the z-dependent probe field amplitude, n is the fiber refractive index, c is the light velocity in the vacuum, and $g_{B,ac}$ is the ac Brillouin gain given by

(7) $$g_{B,ac}\left(z,\omega_{m}\right)=\frac{\eta_{SBS}{\left(z\right)g}_{B0}}{2}\left(\frac{1}{1-j\frac{\Delta \left(z\right)}{\Gamma_{1}}}+\frac{1}{1+j\frac{\Delta \left(z\right)}{\Gamma_{1}}}+\frac{1}{1-j\frac{\left(\Delta \left(z\right)-\omega_{m}\right)}{\Gamma_{1}}}+\frac{1}{1+j\frac{\left(\Delta \left(z\right)+\omega_{m}\right)}{\Gamma_{1}}}\right)$$

In Equation (7), $g_{B0}$ is the SBS peak gain coefficient, $\Gamma_{1}$ is the acoustic damping rate, and $\Delta \left(z\right)=2\pi \left(\Delta_{\nu pp}-\nu_{B}\left(z\right)\right)$ is the z-dependent detuning, i.e., the difference between the pump–probe frequency shift $\Delta_{\nu pp}$ and the local Brillouin frequency shift $\nu_{B}$. Equations (5)–(7) reveal that any spatial variation of the SBS mixing coefficient results in a spatial modulation of the Brillouin gain, even in absence of temperature- or strain-related $\nu_{B}$ spatial changes [17]. Following this, it can be argued that the measurement of the Brillouin peak gain distribution in a spun fiber may provide insight into the birefringence-induced SOP variations of the interacting beams, provided that such measurement is realized with sufficient spatial resolution. In BOFDA measurements, the spatial resolution is given by [15]

(8) $$\Delta z=\frac{c}{2n}\frac{1}{f_{m,max}-f_{m,min}}$$

where $f_{m,max}$ and $f_{m,min}\;$denote the maximum and minimum modulation frequencies, respectively. In other words, the BOFDA spatial resolution is determined by the frequency range adopted for the measurement of the baseband transfer function TF.

**Figure 5:**
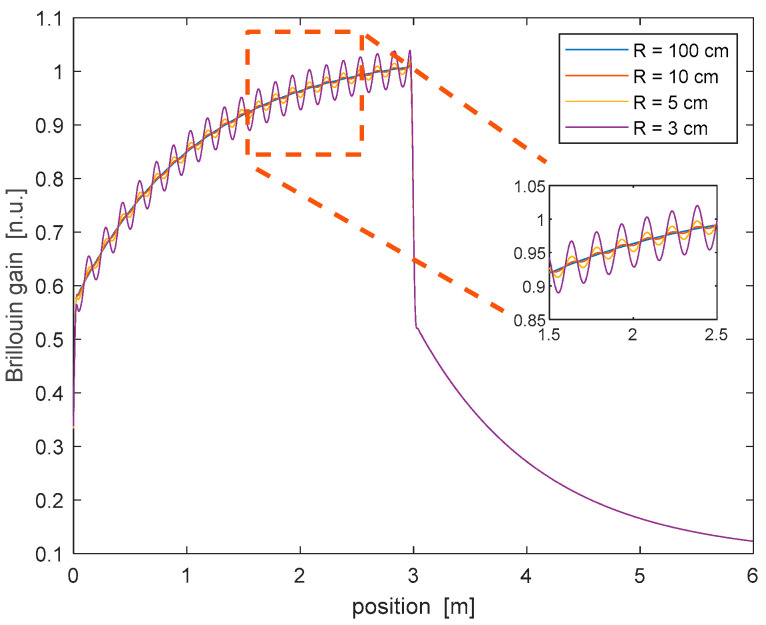
Brillouin gain profile computed for a spun fiber with *L* = 3 m, at varying curvature radii.

The presented theory was applied to calculate the Brillouin gain distribution along a spun fiber with L=3 m, $L_{S}=4\;mm,$ and $L_{b}=12.2\;mm$. Specifically, Equation (2) was numerically solved to determine the SOP evolution of the pump and probe beams. The SOP trajectories were then employed to calculate the Brillouin gain response at a spatial resolution of 31 mm. As the latter contains many spin periods, we could safely neglect the SOP-dependence of $\nu_{B}$ arising from the linear birefringence of the unspun fiber [6]. We also assumed that $\nu_{B}$ was constant along the fiber (uniform temperature and strain conditions). Figure 5 shows the Brillouin gain profile computed using the same curvatures adopted in Figure 4. The results revealed that, for a large curvature radius (R = 100 cm), no undulation is visible in the simulated trace. In these conditions, the Brillouin gain follows the typical response of a BOFDA sensor, characterized by a first rapid increase (or decrease) dictated by the spatial resolution, followed by a slow transient determined using the photon lifetime [18]. For lower curvatures radii, the Brillouin gain acquires a periodic undulation with a period equal to$\;L_{C}\;$(15 cm in our simulations), growing in amplitude at tighter bending. This result can be explained by considering that, in a straight spun fiber (or a bent spun fiber with a large curvature radius), the SOPs of the pump and probe beams remain almost linear during their propagation, with their azimuth varying linearly along z with the same slope (see Figure 3). In such conditions, their azimuth angle difference will be constant, except from rapid oscillations at a period of 2 mm. As the SBS mixing efficiency is roughly determined using the cosine of their azimuth angle difference (see Equation (5)), no polarization-induced oscillation will be impressed on the Brillouin gain in these conditions, also because of the limited spatial resolution adopted for the BOFDA simulations (i.e., $\Delta z\gg L_{S})$. For a tighter radius bend, instead, the two quasi-circular eigenmodes couple each other [13], leading to a slow undulation of the azimuth and ellipticity angles, as shown in Figure 4. These undulations are impressed on the Brillouin gain through the SBS mixing efficiency.

**Figure 6:**
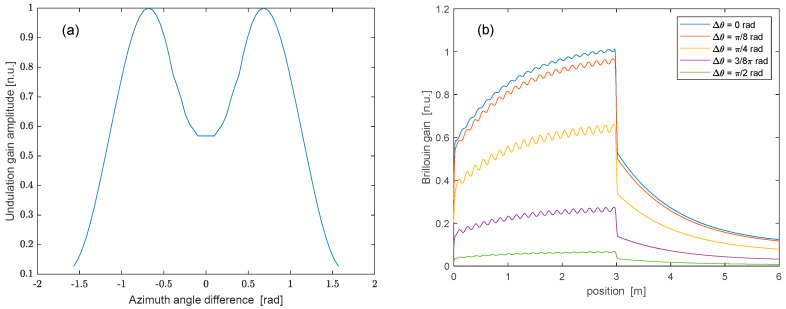
(**a**) Amplitude of the Brillouin gain undulations in a bent spun fiber with R=3 cm, as a function of the difference between the azimuth angles of the input SOP of pump and probe waves; (**b**) Brillouin gain profile along a spun fiber with L=3 m, as computed using the same parameters adopted for calculating the SOP trajectories shown in Figure 4.

As a final remark, it is worth investigating if, for a fixed curvature, the amplitude of the Brillouin gain undulations can be maximized by properly choosing the input SOP of the pump and probe waves. To this aim, we repeated the simulation while varying the azimuth angle difference between the input SOP of the pump and probe fields, from $-\frac{\pi }{2}$ to $+\frac{\pi }{2}$. The ellipticity angle (i.e., the latitude on the Poincaré sphere) of both waves was kept fixed, and equal to the ellipticity of the sum of the two eigenmodes, i.e., ϵ=−0.08 rad (see Figure 4b). The results of this analysis are summarized in Figure 6a, which shows the normalized amplitude of the gain undulation as a function of the azimuth angle difference between the pump and probe waves, for a fixed radius of curvature R=3 cm. As anticipated, the maximum undulation occurred for an azimuth angle difference of π/4 rad. For clarity, we show in Figure 6b the Brillouin gain profile calculated for a discrete set of azimuth angle differences. We observe that, while the maximum dc gain occurs for Δθ=0 rad, the maximum undulation amplitude is achieved for Δθ=π/4rad.

In conclusion, our numerical analysis suggests that, in a bent spun fiber with a curvature radius equal to or less than ≈10 cm, the Brillouin gain spatially oscillates with a period equal to $L_{C}$. Thus, the latter can be retrieved using conventional SBS measurements, provided that the latter are performed with a sufficient spatial resolution (i.e., $\Delta z\ll L_{C}$).

## 3. Experimental Results

**Figure 7:**
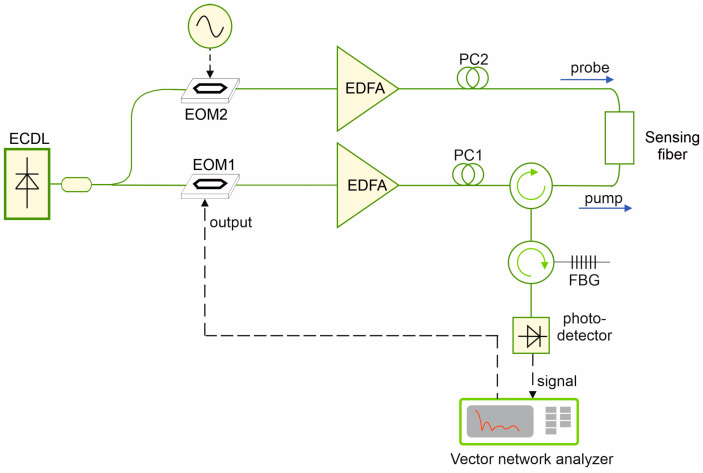
Experimental setup for BOFDA measurements. ECDL: External-cavity diode laser; EDFA: Erbium-doped fiber amplifier; PC: polarization controller; EOM: electro-optic intensity modulator; FBG: fiber Bragg grating.

A number of experimental tests were carried out using the BOFDA setup shown in Figure 7. In brief, the output of a distributed feedback laser diode (PPCL500, Pure Photonics, Milpitas, CA, USA) operating at λ=1550 nm was split into a pump and a probe branch. On the pump branch, a small sinusoidal modulation was impressed to the light by an electro-optic modulator (EOM1), biased at quadrature and driven by the output port of a VNA (M9374A, Keysight, Santa Rosa, CA, USA). The modulated pump wave was then amplified using an erbium-doped fiber amplifier (EDFA), passed through a manual polarization controller (PC1), and injected into the FUT. On the probe branch, the light was double sideband modulated (DSB) using another electro-optic intensity modulator (EOM2) biased at null point and driven by a microwave generator (SynthHD, Windfreak Technologies, New Port Richey, FL, USA). The modulated light was amplified through another EDFA, passed through another manual polarization controller (PC2), and finally injected into the opposite end of the spun fiber. The transmitted probe light was first filtered using a narrowband (≈4 GHz) fiber Bragg grating (FBG) to suppress the lower sideband generated by EOM2, and then detected using a 12 GHz photodetector (Model 1544, New Focus, Irvine, CA, USA) connected to the input port of the VNA. All the measurements shown in this paragraph were carried out at a spatial resolution of 31 mm, i.e., sweeping the VNA frequency up to 3.2 GHz. The manual polarization controllers PC1 and PC2 were adjusted each time to maximize the Brillouin gain undulations.

The fiber used for our experiments was a 3 m long spun fiber (SHB1500 by Fibercore, Las Vegas, NV, USA), fabricated by spinning a bow-tie style polarization-maintaining preform during the drawing process. The fiber had an attenuation of ≈0.33 dB/km at λ = 1550 nm and a spin period of $L_{S}=4\;mm$. The nominal elliptical beat length $L_{C}$ of the fiber spool was 15 cm, from which we inferred a linear beat length of the unspun fiber $L_{b}=12.2\;mm$ (see Equation (3)), the same used for simulations.

**Figure 8:**
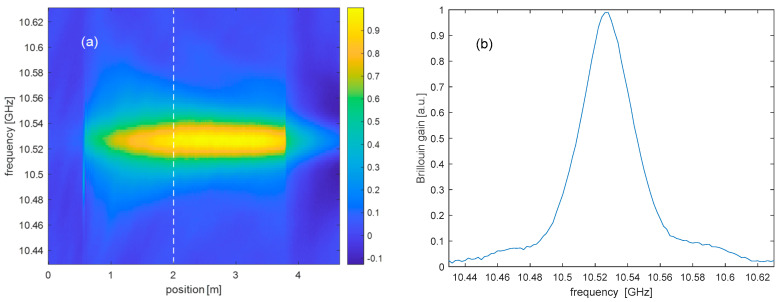
(**a**) Brillouin gain map acquired along the straight spun fiber; (**b**) Brillouin gain spectrum acquired at z = 2 m, i.e. at the position indicated by a white dashed line in (**a**).

We show in Figure 8a the Brillouin gain map acquired along the straight spun fiber (no bending), varying the pump–probe frequency shift from 10,430 MHz to 10,630 MHz. In this case, the Brillouin gain does not exhibit any evidence of undulations. By extracting the Brillouin gain spectrum at z = 2 m (dashed vertical line in Figure 8a), we recover a Brillouin frequency shift $\nu_{B}=10,527\;MHz$ and a full-width-at-half-maximum $\Delta \nu_{B}\approx 37\;MHz$ (see Figure 8b).

**Figure 9:**
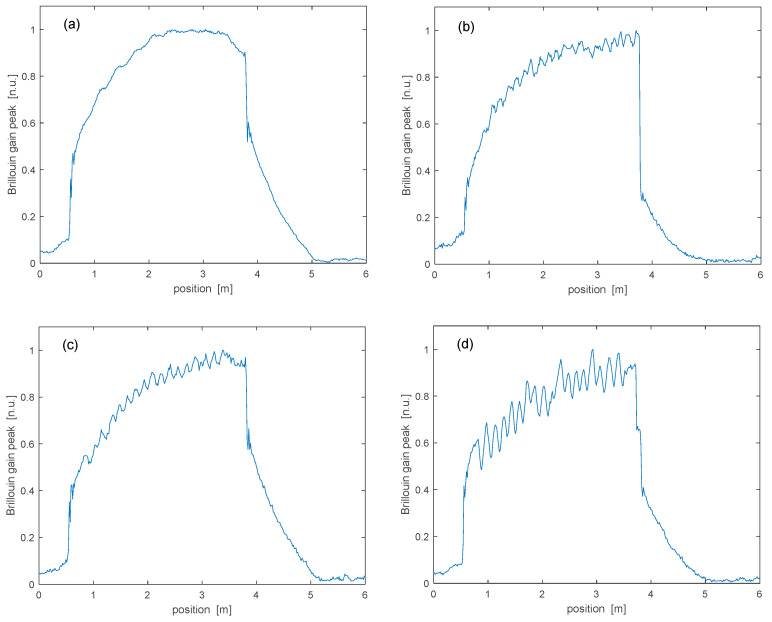
Brillouin gain peak as a function of fiber position, for different curvature radii: (**a**) R = 7.5 cm; (**b**) R = 4 cm; (**c**) R = 3 cm; (**d**) R = 1.75 cm.

As a next step, the Brillouin gain map was acquired after winding the spun fiber around spools with different curvature radii. We show in Figure 9 the Brillouin gain coefficient obtained by extracting the BGS peak at each sensed position, for four different curvature radii (R = 7.5 cm, R = 4 cm, R = 3 cm, R = 1.75 cm). The figure confirms that, when the spun fiber is subject to bending, the Brillouin gain acquires a slow undulation, with an amplitude growing with decreasing curvature radii. Note that the curvature radius was not decreased further to avoid a significant increase in the bending loss.

By analyzing the gain undulations in the frequency domain, an average spatial period of 14.8 ± 0.3 cm was estimated, which is consistent with the nominal elliptical birefringence beat length of our spun fiber.

**Figure 10:**
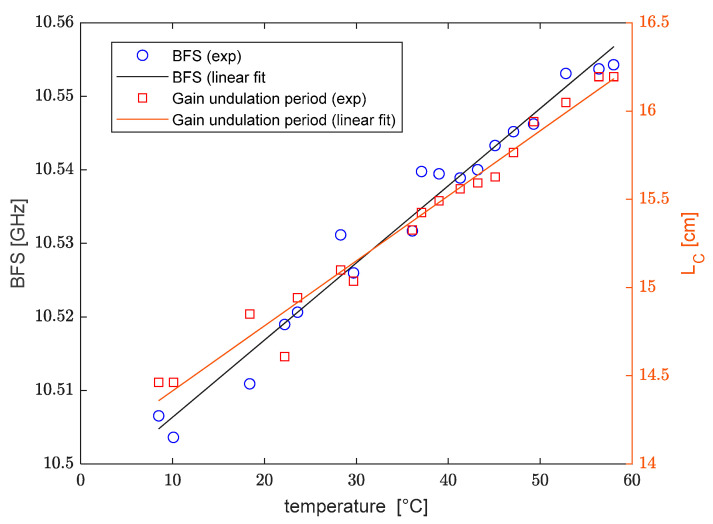
Brillouin frequency shift (blue circles) and Brillouin gain undulation period (red squares) as a function of the temperature, together with the respective linear fits.

As a final step, we analyzed the temperature and strain sensitivity of the Brillouin frequency shift and elliptical birefringence of our spun fiber. For temperature characterization, the fiber was first coiled with a radius of curvature R=3 cm and then immersed in a water bath at varying temperatures. The results are presented in Figure 10. In particular, the figure shows the BFS obtained by applying a conventional Lorentzian fit to the acquired spectra, and, on a different axis, the period of the gain undulation (i.e., the estimated elliptical beat length). Applying a linear fit to our experimental results, a BFS temperature sensitivity $C_{T,\nu_{B}}=1.05\pm 0.09\;MHz/{}^{\circ}C$ was retrieved, which is consistent with the typical BFS sensitivity of silica fibers. The Brillouin gain undulation period shows a temperature sensitivity $C_{T,BL}=368\;\pm \;31\;µm/{}^{\circ}C$. In normalized units, this corresponds to a relative sensitivity of 0.25 ± 0.02%/°C. The latter is in reasonable agreement with the value obtained experimentally in Ref. [19] for a slightly different spun fiber (Fibercore SHB1250) using binary polarization rotators (0.152%/°C). As a further confirmation, we can use Equation (3) to estimate the temperature sensitivity of $L_{C}$ based on the temperature sensitivity of $L_{B}$ and $L_{s}$. As regards $L_{B}$, a temperature sensitivity of −0.13 %/°C was derived experimentally in Ref. [6] using BDG, while for $L_{S}$ we made use of the linear expansion coefficient of silica glass ($0.5\cdot 10^{-6}{\;{}^{\circ}C}^{-1}$ [20]). Putting these values together, Equation (3) permits us to derive a temperature sensitivity of $L_{C}$ equal to 0.25 %/°C, in surprising agreement with our experiments.

**Figure 11:**
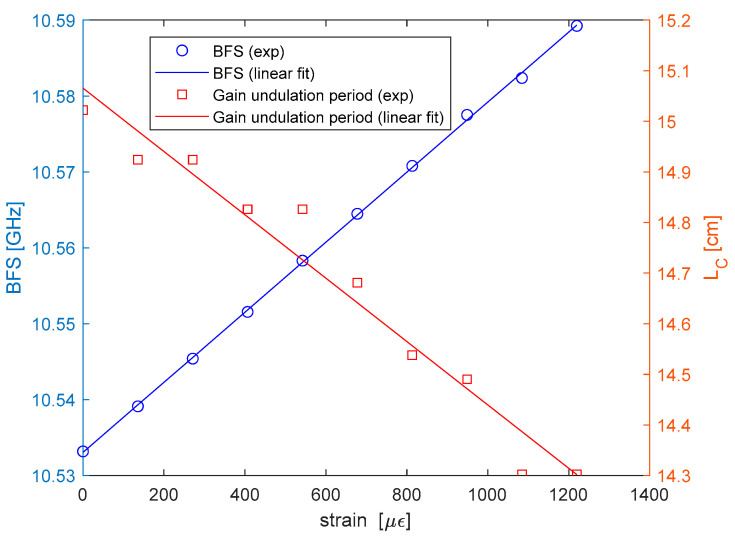
Brillouin frequency shift (blue circles) and Brillouin gain undulation period (red squares) as a function of the strain, together with the respective linear fits.

A similar analysis was carried out to determine the strain dependence of $L_{C}$. For strain characterization, the fiber was wound around two pillars placed at distance of ≈5 cm, one fixed and another one movable through a micro-positioner; in this way, an increasing tensile strain was applied to the spun fiber by moving the micro-positioner. The results are shown in Figure 11. From the linear fit, we derived a BFS/strain sensitivity of $C_{\varepsilon ,\nu_{B}}=46\pm 1\;kHz/µ\varepsilon$ (as typical for silica glass fibers), and an elliptical beat length strain sensitivity of $C_{\varepsilon ,BL}=-6.26\pm 0.1\;\mu m/\mu \varepsilon$. In percentage terms, the latter corresponds to a sensitivity of $-4.17\times 10^{-3}\;\pm \;0.7\times 10^{-3}\;\%/µ\varepsilon .\;$To check the consistency of this value, we can still refer to Equation (3): for $L_{b}$, using the strain sensitivity experimentally determined using BDG in a PANDA fiber (2.04 × 10^−3^ %/µε^−1^ [6]) and accounting for the strain-induced variation of the spin pitch $L_{s}$, Equation (3) permits us to derive a strain sensitivity for $L_{C}$ equal to ≈$-4.15\times 10^{-3}\;\%/µ\varepsilon .$, which is in good agreement with our experimental findings.

Our experimentally determined sensitivities can be used to estimate the uncertainty of simultaneous temperature and strain measurements [21]. Assuming a typical $\nu_{B}$ measurement uncertainty of ±1 MHz and an $L_{C}$ uncertainty of ±1 mm, the resulting temperature and strain uncertainty are ±2.2 °C and ±63 µε, respectively, which may be acceptable for selected applications.

**Figure 12:**
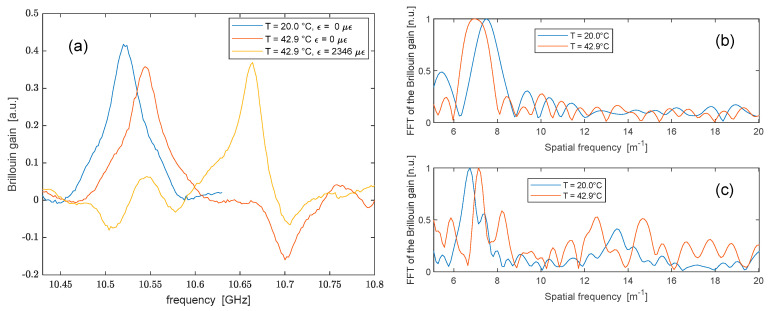
(**a**) BGS acquired at the middle of the free coil (blue and red curves) or the middle of the strained coil (yellow coil); (**b**) Fast Fourier transform of the Brillouin gain undulations measured along the free coil; (**c**) Fast Fourier transform of the Brillouin gain undulations measured along the strained coil.

As a final experiment aimed at assessing the capability of the proposed method of measuring the strain and temperature simultaneously, we measured the $\nu_{B}$ and $L_{C}$ changes over two 1 m long spun fiber coils, both immersed in a water bath. One coil was free, while the other was wrapped around a full water bottle. In this way, the second coil was subject to both temperature and strain changes arising from the thermal expansion of the water bottle. Then, we performed two measurements, one at T = 20.0 °C and another one at T = 42.9 °C, with the temperatures provided by a thermocouple immersed in the water bath. In Figure 12a, we show the BGS acquired at the middle of the free coil at T = 20.0 °C (blue curve) and T = 42.9 °C (red curve), as well as the BGS acquired at the middle of the strained coil at T = 42.9 °C (yellow curve). As expected, the BFS at the strained position is the highest, as it is influenced by both strain and temperature changes. Note that the strain value reported in the legend (ε=2346 με) was derived based on the measured $\nu_{B}$ shift and the previously determined BFS strain sensitivity ($C_{\varepsilon ,\nu_{B}}=46\;kHz/µ\varepsilon$). Using the same measurements, we could also estimate the elliptical beat length variation, based on the period of the Brillouin gain peak undulations. We show in Figure 12b,c the Fast Fourier Transforms (FFT) of the Brillouin gain undulations windowed along either the free coil or the strained coil. We noted that the peaks associated with the SOP-induced gain undulations are quite broad, due to the limited number (≈6) of periods along the coil length, and due to the variations in the BFS along the same coil (the latter were especially evident along the strained coil). Nonetheless, the acquired traces allowed us to estimate the BFS change, $∆\nu_{B}$, and the elliptical beat length change, ${\Delta L}_{C}$, when passing from T = 20.0 °C to T = 42.9 °C.

Based on these values, we calculated the temperature and strain changes at the chosen positions through

(9) $$\left(\begin{array}{c} \Delta T\\ \varepsilon \end{array}\right)=M^{-1}\left(\begin{array}{c} \Delta \nu_{B}\\ {\Delta L}_{C} \end{array}\right)$$

where $M=\left(\begin{array}{cc} C_{T,\nu_{B}} & C_{\varepsilon ,\nu_{B}}\\ C_{T,BL} & C_{\varepsilon ,BL} \end{array}\right)$ is the matrix of the previously determined coefficients. We summarize in Table 1 the results obtained applying Equation (9) for the two positions. The obtained values of ΔT at both positions are in good agreement with the thermocouple reading (22.9 °C). With regard to the strain, the obtained values differ from their nominal values (0 µε along the free coil and ε=2346 µε along the strained coil) by less than the previously determined strain uncertainty.

## 4. Conclusions

In this paper, we experimentally verified that the Brillouin gain in bent spun fibers exhibits a periodical undulation, with a period equal to the elliptical beat length of the spun fiber. Therefore, the birefringence of the fiber can be recovered using conventional Brillouin measurements. The mechanism leading to the formation of these undulations was analyzed by numerically studying the SOP evolution in bent spun fibers. We note that, while previous works had analyzed the mode coupling induced by bending in silica [22] and plastic [23] fibers, this is the first study that has analyzed the influence of bending on the Brillouin gain response in spun optical fibers. We have shown that spun fibers could be used for simultaneous strain and temperature measurements, owing to the simultaneous dependence of the BFS and elliptical beat length from these measurands. The main limitation is likely to be the necessity of bending the fiber to make these gain undulations visible. This may restrict the use of these fibers in specific applications in which the fiber can be installed in a bent configuration, e.g., using a spiral winding layout in pipeline monitoring [24], or wrapped around a pressure vessel [25]. Further work is also necessary to verify the capability of the proposed methodology for measuring the magnetic fields through Faraday rotation [7].

## Figures and Tables

**Table 1:** Experimental values of ${∆\nu }_{B}$ and ${∆L}_{C}$ when passing from T = 20.0 °C to T = 42.9 °C, and corresponding temperature and strain changes calculated using Equation (9).

	Free Coil		Strained Coil	
$\Delta \nu_{B}$ [MHz]	21.8		129.7	
${\Delta L}_{C}$ [mm]	8.8		−5.7	
ΔT [°C]	22.9 (actual)	23.1 (meas)	22.9 (actual)	22.7 (meas)
ε [µε]	0 (actual)	−54 (meas)	2346 (actual)	2301 (meas)

## Data Availability

The original contributions presented in this study are included in the article. Further inquiries can be directed to the corresponding author.
